# The Association Between the Use of Low-Slice Computed Tomography Machines and Downstream Care: Comparative Study of 16-Slice and 64-Slice Computed Tomography Angiography

**DOI:** 10.2196/32892

**Published:** 2022-06-30

**Authors:** Adam C Powell, James W Long, Uday U Deshmukh, Jeffrey D Simmons

**Affiliations:** 1 HealthHelp Houston, TX United States; 2 Humana Inc Louisville, KY United States

**Keywords:** computed tomography, tomography, diagnostic imaging, outpatient, angiography, obsolescence, computed tomography angiography of the neck, neck, low-slice computed tomography, cervicocerebral angiography, downstream testing, computed tomography machine, invasive testing, machine, testing, invasive

## Abstract

**Background:**

Although computed tomography (CT) studies on machines with more slices have reported higher positive and negative predictive values, the impact of using low-slice (16-slice) CT machines on downstream testing has not been well studied. In community outpatient settings, low-slice CT machines remain in use, although many hospitals have adopted higher-slice machines.

**Objective:**

This study examines the association between the use of low-slice CT machines and downstream invasive testing in the context of the CT angiography of the neck.

**Methods:**

Included health insurance claims pertained to adults with commercial or Medicare Advantage health plans who underwent the CT angiography of the neck. Site certification data were used to assign counts of slices to claims. Claims that were made in the 60 days after CT were examined for cervicocerebral angiography. The association between the number of slices and cervicocerebral angiography was evaluated by using a chi-square test and multivariate logistic regression.

**Results:**

Claims for 16-slice CT had a 5.1% (33/641) downstream cervicocerebral angiography rate, while claims for 64-slice CT had a 3.1% (35/1125) rate, and a significant difference (*P*=.03) was observed. An analysis that was adjusted for patient demographics also found a significant relationship (odds ratio 1.64, 95% CI 1.00-2.69; *P*=.047).

**Conclusions:**

The use of low-slice CT machines in the community may impact the quality of care and result in more downstream testing.

## Introduction

Although there have been great advances in computed tomography (CT) technology, when undergoing outpatient imaging in the community, many patients continue to have imaging performed at underresourced facilities with a single, often low-slice CT machine. Further, while high-slice CT machines with 128 detector rows (slices) or more are often available at academic medical centers, in the community, 16-slice CT (low-slice CT) machines remain in active use, and the use of 64-slice CT (medium-slice CT) machines is common. This study aims to explore whether the use of low-slice CT at some facilities has the potential to impact patient outcomes.

As CT machines have advanced, systems with increasing numbers of detector rows have become available. In 2004, the first clinical images from a 64-slice CT machine were released to the public, and a machine was commercially launched in the American market [1]. In part due to the increased costs of 64-slice CT machines, their adoption has not been universal. By 2016—over 1 decade after 64-slice CT became available—only 63% of hospitals had access to a CT machine with 64 or more slices [2]. Thus, a substantial number of patients continue to undergo CT on machines with fewer slices. Outpatient facilities with a single CT machine may not have medium-slice or high-slice machines, as they may lack the volumes necessary for justifying their purchase. Nonetheless, the use of low-slice machines may impact patients’ care, and facilities that are only able to offer 16-slice CT may wish to consider upgrading if the use of such technology has an impact on downstream care.

The benefits of 64-slice CT, relative to 16-slice CT, have primarily been examined in terms of the quality of visualization, positive predictive value, negative predictive value, sensitivity, and specificity [3-6]. However, researchers have not yet examined the association between the use of 64-slice and 16-slice CT machines and downstream testing. This study provides preliminary insights into the association between the number of slices and downstream testing in a narrow context to explore whether an association may exist. It has historically been difficult to study how the type of CT machine used impacts downstream testing within the context of a broad population because health insurance claims data—the main source of information for this type of study—do not provide any information about the CT machine used to acquire an image. Although electronic medical records may contain relevant information, these data may be challenging to use when answering this question because patients may undergo testing at multiple facilities, thereby creating issues with linking CT scans to downstream testing. Furthermore, health care providers may not have ample data related to both 16-slice and 64-slice CT, as providers typically own a small number of CT machines (if they have more than 1 CT machine), thereby hampering comparisons between the two types of machines.

The better the information that can be gathered from a CT machine, the greater the extent to which it may be a substitute for other forms of testing. Current evidence suggests that as CT technology advances, the potential to replace coronary angiography for the evaluation of coronary artery disease with CT scans increases [7]. If higher-quality CT imaging can reduce the use of angiography, it has the potential to improve the welfare of patients, as angiography can result in complications. A review of 19,826 patients who underwent diagnostic cerebral angiography found that neurological complications occurred in 2.63% of patients, with 0.14% experiencing strokes resulting in permanent disability [8].

In order to assess the potential benefits of the use of CT machines with a greater number of slices in underresourced outpatient settings, this study examined the association between the use of 16-slice and 64-slice CT machines to perform the CT angiography of the neck and subsequent cervicocerebral angiography within a population of patients that had not recently undergone head/neck imaging, catheterization, or percutaneous coronary interventions and had undergone imaging at facilities with only a single type of CT machine. If patients who underwent CT via a 64-slice machine were less likely to undergo subsequent cervicocerebral angiography than those who underwent CT via a 16-slice machine, then there may be quality benefits to 64-slice CT, in addition to the visual and informational benefits that have previously been characterized [3-6]. If the use of low-slice CT machines impacts the quality of care that patients receive, facilities may need to consider upgrading their equipment, and ordering physicians may need to more closely consider the capabilities of the equipment that is present at the imaging facilities to which they make referrals. Although low-slice CT machines remain in use in the community, used medium-slice and high-slice CT machines are readily available on the secondary market, and many facilities offer superior forms of CT.

## Methods

### Data Source and Sample Population

All health insurance claims for the CT angiography of the neck (current procedural terminology code: 70498) with dates of service ranging between September 15, 2017, and September 14, 2018, were extracted from the database of a national health care organization. Health insurance claims pertained to adults with commercial and Medicare Advantage health plans. The dates of the CT scans served as the index dates. Claims were excluded from this study if they pertained to patients who were not continuously enrolled in their health plan from 90 days prior to the index date to 60 days following the index date. To restrict this study to patients beginning new episodes of care, claims were likewise excluded if they pertained to patients who had received a CT image, magnetic resonance image, or positron emission tomography image of the head or neck in the 90 days prior to the index date or if they pertained to patients who had undergone catheterization or a percutaneous coronary intervention in the same time period. Claims could only be linked to a CT machine with a known number of slices if CT was performed with a CT machine that was based at a facility participating in an outpatient site of a service certification program and if the site of service had either 1 CT machine or multiple CT machines with the same number of slices. Claims were excluded from this study if they could not be matched to a CT machine with a known number of slices. Finally, claims were excluded if they pertained to a CT machine with a number of slices other than 16 or 64.

### Ethics Approval

This study was reviewed and approved by Advarra’s institutional review board (approval number: Pro00033618). The institutional review board granted a waiver of informed consent for this study due to its aggregate, observational nature. This study was conducted in accordance with the Declaration of Helsinki.

### Measurement

The dependent variable in the analysis was whether a CT claim was followed by a claim for cervicocerebral angiography (current procedural terminology codes: 36221-36228) within 60 days. The independent variable was whether a CT image was acquired via a 16-slice CT machine or a 64-slice CT machine. The covariates that were included as potential confounders in the analysis were variables for whether the patients pertaining to the claims lived in areas with above or below the median income of the sample, as well as their sex, urbanicity, health plan’s line of business (commercial vs Medicare), and age (<65 years vs ≥65 years). The average income within a patient’s zip code was determined by using the median income of the past 12 months in 2017 inflation–adjusted US dollars, as reported by the American Community Survey [9]. Rural areas were identified by using a zip code mapping table that was developed by the Centers for Medicare & Medicaid Services [10].

### Analysis

Descriptive statistics were calculated for the sample. Chi-square tests were conducted to assess whether the population that underwent 16-slice CT differed from the population that underwent 64-slice CT on each of the potential confounders. Chi-square tests were also conducted to test for a univariate association between each of the variables within the study and the outcome—whether a patient underwent downstream cervicocerebral angiography. A multivariate logistic regression was conducted to determine the adjusted association between the number of slices that the used CT machine possessed and downstream cervicocerebral angiography. The results from the multivariate logistic regression were reported as odds ratios. Throughout the analysis, a *P* value of <.05 was used as the threshold for determining statistical significance.

## Results

**Figure 1:**
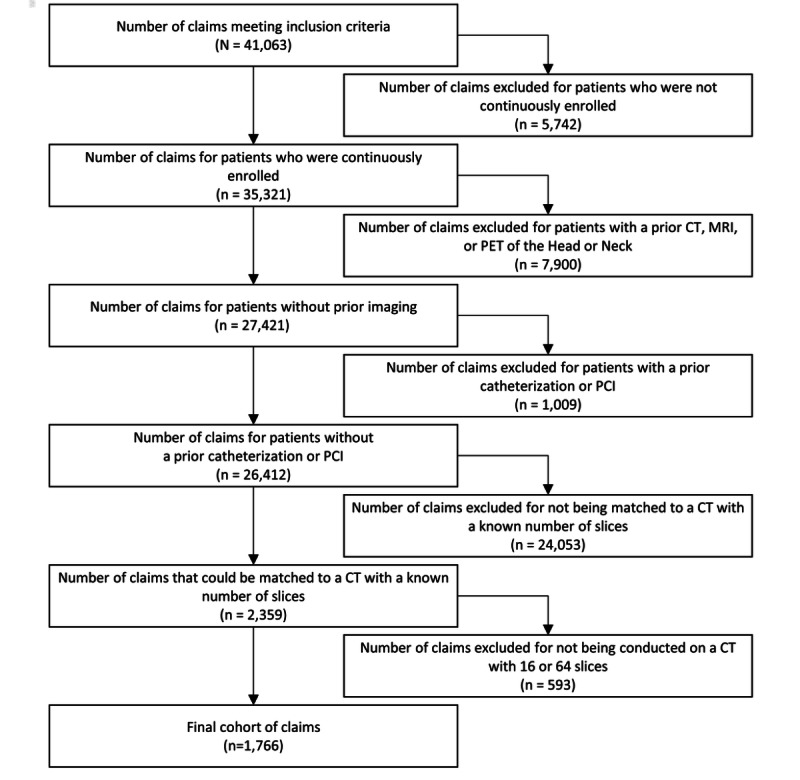
Sample selection diagram. CT: computed tomography; MRI: magnetic resonance imaging; PCI: percutaneous coronary intervention; PET: positron emission tomography.

As shown in Figure 1, of the 41,063 claims that met the initial inclusion criteria, 1766 remained qualified for this study after the consideration of the exclusion criteria. Many of the exclusions occurred due to situations in which it was infeasible to match claims to specific CT machines. The claims occurred at 252 different rendering facilities, of which 121 had 16-slice CT machines and 131 had 64-slice CT machines. Descriptive statistics are presented in Table 1. Among the total cohort, 36.3% (641/1766) of claims were related to CT that was performed with a 16-slice machine, while the majority were related to CT that was performed with a 64-slice machine (1125/1766, 64.7%). The patients who underwent CT had a mean age of 71 years and were from communities with a mean local income of US $57,460. Most of the claims pertained to patients who had Medicare Advantage plans (1620/1766, 91.7%); only 8.3% (146/1766) pertained to patients who had commercial insurance. There was a significant association between community income and the number of slices in the CT machine used (*P*<.001); 46.3% (297/641) of claims for a 16-slice CT came from a community with below the median income, while 37.2% (418/1125) of claims for a 64-slice CT came from a community with below the median income.

**Figure 2:**
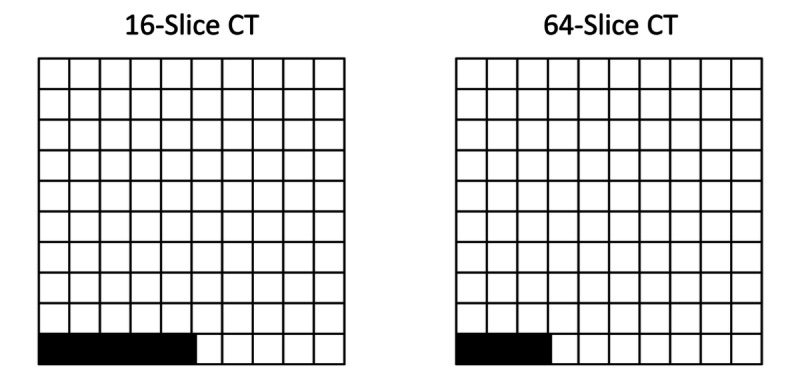
Visual depiction of the downstream cervicocerebral angiography rate following 16-slice CT or 64-slice CT. Each cell represents 1 claim for CT. Shaded cells represent the proportion of CTs that were followed by downstream cervicocerebral angiography. CT: computed tomography.

As shown in Table 2, chi-square tests found that none of the control variables had a significant univariate association with downstream cervicocerebral angiography. However, a chi-square test found that there was a statistically significant univariate relationship between the performance of CT via a 16-slice machine or 64-slice machine and subsequent cervicocerebral angiography (*P*=.03). Claims for 16-slice CT had a 5.1% (33/641) subsequent cervicocerebral angiography rate, while claims for 64-slice CT had a 3.1% (35/1125) subsequent cervicocerebral angiography rate (Figure 2).

The adjusted analysis, which is shown in Table 3, found that there was a significant association between the performance of CT via a 16-slice machine or 64-slice machine and subsequent cervicocerebral angiography (*P*=.047; odds ratio 1.64, 95% CI 1.00-2.69). None of the control variables in the adjusted analysis had a significant or near-significant association with cervicocerebral angiography.

**Table 1:** Descriptive statistics.

Patient characteristic	All claims (N=1766), n (%)	Claims for 16-slice CT^a^ (n=641), n (%)	Claims for 64-slice CT (n=1125), n (%)	*P* value
Below median income (vs above median income or omitted)	715 (40.5)	297 (46.3)	418 (37.2)	<.001
Male (vs female)	844 (47.8)	310 (48.4)	534 (47.5)	.72
Rural (vs urban)	260 (14.7)	93 (14.5)	167 (14.8)	.85
Commercial (vs Medicare)	146 (8.3)	34 (5.3)	112 (10)	.001
Aged under 65 years (vs ≥65 years)	304 (17.2)	96 (15)	208 (18.5)	.06

^a^CT: computed tomography.

**Table 2:** Univariate associations between variables and downstream cervicocerebral angiography.

Patient characteristic	All claims (N=1766), n (%)	Claims not pertaining to cervicocerebral angiography (n=1698), n (%)	Claims pertaining to cervicocerebral angiography (n=68), n (%)	*P* value
Below median income (vs above median income or omitted)	715 (40.5)	685 (40.3)	30 (44.1)	.53
Male (vs female)	844 (47.8)	812 (47.8)	32 (47.1)	.90
Rural (vs urban)	260 (14.7)	252 (14.8)	8 (11.8)	.48
Commercial (vs Medicare)	146 (8.3)	142 (8.4)	4 (5.9)	.47
Aged under 65 years (vs ≥65 years)	304 (17.2)	296 (17.4)	8 (11.8)	.23
16-slice CT^a^ (vs 64-slice CT)	641 (36.3)	608 (35.8)	33 (48.5)	.03

^a^CT: computed tomography.

**Table 3:** Adjusted odds ratios from the multivariate logistic regression.

Patient characteristic	Odds ratio	95% CI	*P* value
Below median income (vs above median income)	1.17	0.70-1.93	.55
Male (vs female)	0.97	0.60-1.59	.92
Rural (vs urban)	0.71	0.30-1.45	.38
Commercial (vs Medicare)	1.05	0.27-3.37	.94
Aged under 65 (vs ≥65 years)	0.63	0.23-1.42	.31
16-slice CT^a^ (vs 64-slice CT)	1.64	1.00-2.69	.047

^a^CT: computed tomography.

## Discussion

### Principal Findings

Compared to patients who had their neck CT angiography performed with a 16-slice machine, patients who had their neck CT angiography performed with a 64-slice machine were significantly (*P*=.03) less likely to undergo a subsequent cervicocerebral angiography. If the patients who were referred for 16-slice CT were not believed a priori to be more difficult to conclusively diagnose than those referred to 64-slice CT, the findings suggest that the use of 64-slice CT machines may improve the quality of care by reducing the need for downstream invasive testing. Although they are not universally available, 64-slice CT machines are widely distributed and may be the best choice for patients requiring a CT scan. Considering the data presented herein, physicians may want to review the specifications of the CT machines that a rendering facility possesses when referring their patients.

The findings from this study pertain to care that was delivered in 2017 and 2018—over 1 decade after the introduction of 64-slice CT. Of the 2359 total CT claims that could be tied to a CT machine with a known number of slices (Figure 1), 641 claims were tied to a 16-slice CT (Table 1), representing 27.2% of the total. Although 64-slice CT was performed more frequently than 16-slice CT in the context examined, the findings suggest that 16-slice CT machines remain in active use in many facilities.

The findings of this study are congruent with those of prior research on the benefits of performing CT by using the greatest number of slices available. A previous study found that when a 64-slice CT machine was used to assess left ventricular function, the values it produced were more similar to those obtained via echocardiography and technetium-99m gated single-photon emission CT compared to the values produced by a 16-slice CT machine [11]. A multicenter prospective study on the utility of 16-slice versus 64-slice CT in screening patients for coronary artery disease did not detect significant differences between 16-slice and 64-slice CT in terms of sensitivity, specificity, positive predictive value, or negative predictive value when CT-based findings were compared to coronary angiography–based findings, although it is possible that the study was underpowered [5]. Furthermore, a literature review of the diagnostic performance of 16-slice versus 64-slice CT, in comparison with coronary angiography, found that 64-slice CT had higher sensitivity values, specificity values, positive predictive values, and negative predictive values [4].

Finally, although community income was not a significant (*P*=.55) variable in the adjusted model for estimating the determinants of cervicocerebral angiography, community income has an association with the number of slices that the used CT machine possessed. There may be an access disparity issue wherein patients from lower-income communities are less able to access 64-slice CT. Although evidence of a clinical impact resulting from this was found by this study, the association between income and the nature of the CT machine used may require future investigation.

In our study, we examined 1 current procedural terminology code for neck CT, which represented only a small fraction of the overall CT imaging conducted in the United States. In 2017, America’s 39 million traditional Medicare beneficiaries collectively underwent 16 million CTs, which were billed through their Medicare Part B benefits [12,13]. Millions of other CTs were performed on patients with commercial, Medicaid, and Medicare Advantage health plans. Although not all examinations may benefit from being performed via 64-slice CT rather than 16-slice CT, it is possible that there are other indications for which the benefit of 64-slice CT could be demonstrated. Further research on the downstream consequences of the choice of a CT machine has the potential to impact many people due to the large number of CTs that are conducted in the United States each year.

### Limitations

There are several limitations that need to be considered when interpreting our findings. As this was a claims-based analysis, it is unknown whether patients who underwent 16-slice CT or 64-slice CT differed in terms of factors other than those that were examined. It is possible that clinical differences influenced assignment. For the findings to have been a product of biased assignment, physicians would need to have preferred assigning patients with more ambiguous cases to 16-slice CT, which is a choice that seems counterintuitive.

The findings of this study may not be representative of the care that is delivered to the overall population, as only outpatient facilities with a single CT machine or a set of CT machines with the same number of slices could be included in the analysis. Thus, the sample does not include facilities with a diverse set of CT machines. This requirement forced the exclusion of many of the available claims. If ordering physicians’ choice of a rendering facility is influenced by proximity rather than by the nature of their CT machine, then their choice might serve to counterbalance the potential for assignment bias.

Lastly, the population studied was not representative of the overall population of the United States. The individuals included in this study resided predominantly in the south, as this is where the health care organization that supplied the data had the strongest presence. The sample likewise did not contain anyone with traditional Medicare or anyone with a Medicaid plan lacking dual eligibility for Medicare. As the incomes of the patients in the sample are unknown, the average incomes within their zip codes were used as a proxy. Given the average age of the patients in the sample, it is likely that many were retired and were earning incomes lower than those that were typical for their communities.

### Conclusions

The analysis found a significant association between the performance of CT via a 16-slice CT machine or 64-slice CT machine and subsequent cervicocerebral angiography before (*P*=.03) and after (*P*=.047) adjusting for patient demographic factors. None of the other factors examined had a significant association with subsequent cervicocerebral angiography. When patients can potentially have access to imaging via 64-slice CT, ordering physicians should consider the potential benefits of directing patients to undergo the CT angiography of the neck via a 64-slice CT machine rather than a 16-slice CT machine. Further research is needed to explore the impact of the decision to use low-slice CT machines in additional clinical contexts and examine whether the relationship remains significant after controlling for clinical factors. Although higher-slice CT machines may not be readily available in some communities, our findings suggest that physicians need to weigh the benefits of access against the benefits of having patients undergo a diagnostic examination that is less likely to result in subsequent downstream testing.

## Abbreviations

CT: computed tomography

## References

[1] (2004). Siemens releases first clinical images from 64-slice CT scanner. J Clin Eng.

[2] Percentage of hospitals with access to multi-slice spiral computed tomography: 64 + slice. Harvey L. Neiman Health Policy Institute.

[3] Seifarth H, Ozgün M, Raupach R, Flohr T, Heindel W, Fischbach R, Maintz D (2006). 64- versus 16-slice CT angiography for coronary artery stent assessment: in vitro experience. Invest Radiol.

[4] Hamon M, Morello R, Riddell JW, Hamon M (2007). Coronary arteries: diagnostic performance of 16- versus 64-section spiral CT compared with invasive coronary angiography--meta-analysis. Radiology.

[5] Marano R, De Cobelli F, Floriani I, Becker C, Herzog C, Centonze M, Morana G, Gualdi GF, Ligabue G, Pontone G, Catalano C, Chiappino D, Midiri M, Simonetti G, Marchisio F, Olivetti L, Fattori R, Bonomo L, Del Maschio A, NIMISCAD Study Group (2009). Italian multicenter, prospective study to evaluate the negative predictive value of 16- and 64-slice MDCT imaging in patients scheduled for coronary angiography (NIMISCAD-Non Invasive Multicenter Italian Study for Coronary Artery Disease). Eur Radiol.

[6] Baumüller S, Leschka S, Desbiolles L, Stolzmann P, Scheffel H, Seifert B, Marincek B, Alkadhi H (2009). Dual-source versus 64-section CT coronary angiography at lower heart rates: comparison of accuracy and radiation dose. Radiology.

[7] Khan R, Rawal S, Eisenberg MJ (2009). Transitioning from 16-slice to 64-slice multidetector computed tomography for the assessment of coronary artery disease: are we really making progress?. Can J Cardiol.

[8] Kaufmann TJ, Huston J 3rd, Mandrekar JN, Schleck CD, Thielen KR, Kallmes DF (2007). Complications of diagnostic cerebral angiography: evaluation of 19,826 consecutive patients. Radiology.

[9] S1903: Median income in the past 12 months (in 2017 inflation-adjusted dollars). United States Census Bureau.

[10] (2016). DME-Rural-Zip-and-Formats. Centers for Medicare and Medicaid Services.

[11] Abbara S, Chow BJW, Pena AJ, Cury RC, Hoffmann U, Nieman K, Brady TJ (2008). Assessment of left ventricular function with 16- and 64-slice multi-detector computed tomography. Eur J Radiol.

[12] Computed tomography procedures per 1000 beneficiaries (Medicare Part B by state of residence). Harvey L. Neiman Health Policy Institute.

[13] MDCR ENROLL AB 10. Original Medicare enrollment: Part A and/or Part B enrollees, by age group, calendar years 2012-2017. Centers for Medicare & Medicaid Services.

